# Supplementary material for: The role of export competitiveness in driving Renminbi cross-border settlement: Micro-level evidence from China
Source: PLoS One. 2025 Apr 11;20(4):e0318099. doi: 10.1371/journal.pone.0318099 (PMC11990576; doi:10.1371/journal.pone.0318099)
Supplement: S1 Appendix — (DOCX) [file pone.0318099.s001.docx]

**Table A1.** **Variables definition**

| Variables | Definition of variables | | | |
| --- | --- | --- | --- | --- |
| Benchmark regression variables | | | | |
| *FC* | The sum of the increase in foreign currency receivables and the increase in foreign currency monetary funds to overseas income, with a higher ratio indicating a lower degree of RMB internationalization. | | | |
| *COMP* | Aspects | Variables | Weight(%) | Definition |
|  | resources | Total assets | 12 | Total amount of assets |
|  |  | Proportion of R&D personnel | 16 | the number of R&D personnel to the total number of personnel |
|  | capabilities | Asset-liability ratio | 12 | Total liabilities to total assets |
|  |  | Profitability | 16 | Net profit to operating income |
|  |  | Growth rate of operating income | 15 | Operating income of this year to operating income of last year -1 |
|  |  | Market share | 16 | Business income to that of all enterprises in the same industry |
|  |  | Cost advantage | 13 | Operating income to operating cost |
|  | aggregate | comp | 100 | Weighted summary of the above indicators |
| *AGE* | Sample year minus year of establishment and take logarithms | | | |
| *KL* | Total assets to operating income | | | |
| *TS* | Number of tradable shares to total shares | | | |
| *ROA* | Net profit to total assets | | | |
| *ART* | Operating income to accounts receivable ending balance, and take the square root | | | |
| *CF* | Net cash flows from operating activities to total assets | | | |
| *SOE* | Whether state-owned enterprise dummy variable | | | |
| *ER* | The average USD/CNY exchange rate in the final month of the year | | | |
| *SUB* | Government subsidies to operating income | | | |
| *OI* | The proportion of overseas income | | | |
| Year FE | Year fixed effect | | | |
| Firm FE | Firm fixed effect | | | |
| Robustness test variables | | | | |
| *FC_new* | Increase in foreign currency receivables as a percentage of overseas sales | | | |
| *SELECT* | Whether to enter the sample dummy variable | | | |
| *INV* | The amount of the enterprise's foreign investment and take logarithms | | | |
| *IMR* | inverse Mills ratio, calculated by the two-step Heckman method | | | |
| Mechanism test variables | | | | |
| *ZONE* | Dummy variable for whether the province established free trade zone last year | | | |
| *COMP*ZONE* | Cross-multiplier of the dummy variable for establishment of FTZ and export competitiveness | | | |
| Heterogeneous grouping variable | | | | |
| Firm Size | Total assets above the median is divided into large-firm group, otherwise divided into small-firm group | | | |
| Industry Concentration | Industry Lerner index higher than the median is divided into high industry concentration group, otherwise divided into low industry concentration group | | | |
| Liability Structure | Financial liability ratio higher than the median is divided into financial liability type, otherwise divided into operating liability type | | | |
| Further analyses variables | | | | |
| *DEPEND* | Overseas business costs and take logarithms, the higher the index, the more serious the import dependence of enterprises. | | | |
| *TobinQ* | Market value to total assets, data come from CSMAR | | | |
| *COMP*FC_r* | Cross-multiplier of the inverse of foreign currency settlement share and export competitiveness | | | |
