# Supplementary material for: The role of export competitiveness in driving Renminbi cross-border settlement: Micro-level evidence from China
Source: PLoS One. 2025 Apr 11;20(4):e0318099. doi: 10.1371/journal.pone.0318099 (PMC11990576; doi:10.1371/journal.pone.0318099)
Supplement: Attachment — Submitted filename: PONE-D-24-43217_rev1.pdf [file pone.0318099.s003.pdf]

PLOS ONE

The role of export competitiveness in driving Renminbi cross-border settlement: Micro-level evidence from China

--Manuscript Draft--

**Manuscript Number:**	PONE-D-24-43217
**Article Type:**	Research Article
**Full Title:**	The role of export competitiveness in driving Renminbi cross-border settlement: Micro-level evidence from China
**Short Title:**	Export competitiveness and Renminbi cross-border settlement
**Corresponding Author:**	Lin JIANG<br>Southwest University of Finance and Economics: Southwestern University of Finance and Economics<br>Chengdu, 请选择 CHINA
**Keywords:**	export competitiveness; RMB cross-border settlement; RMB internationalization；export business
**Abstract:**	Cross-border trade settlement in Renminbi (RMB) is a crucial component of China's efforts to internationalize its currency. This study develops micro-level indicators of RMB cross-border settlement to empirically investigate the role of export competitiveness in promoting the use of RMB in international trade. The findings reveal that export competitiveness significantly enhances RMB cross-border settlement, with the establishment of Free Trade Zones further amplifying this effect. Heterogeneity analysis indicates that the impact is more pronounced in industries with higher concentration or in firms with greater operating liabilities. Moreover, the increased use of RMB in cross-border settlements, driven by export competitiveness, is associated with reduced import dependence and enhanced firm value. These results contribute to a deeper understanding of RMB internationalization through the lens of firm-level trade activities and offer valuable empirical insights for the strategic promotion of RMB internationalization.
**Order of Authors:**	Danlu Bu
	Lin JIANG
**Additional Information:**	
**Question**	**Response**
**Financial Disclosure**<br><br>Enter a financial disclosure statement that describes the sources of funding for the work included in this submission. Review the submission guidelines for detailed requirements. View published research articles from *PLOS ONE* for specific examples.<br><br>This statement is required for submission and **will appear in the published article** if the submission is accepted. Please make sure it is accurate.	The author(s) received no specific funding for this work.

Powered by Editorial Manager® and ProduXion Manager® from Aries Systems Corporation

**Funded studies**<br>Enter a statement with the following details:<br>• Initials of the authors who received each award<br>• Grant numbers awarded to each author<br>• The full name of each funder<br>• URL of each funder website<br>• Did the sponsors or funders play any role in the study design, data collection and analysis, decision to publish, or preparation of the manuscript?<br><br>Did you receive funding for this work?	
**Competing Interests**<br><br>Use the instructions below to enter a competing interest statement for this submission. On behalf of all authors, disclose any competing interests that could be perceived to bias this work—acknowledging all financial support and any other relevant financial or non-financial competing interests.<br><br>This statement is required for submission and **will appear in the published article** if the submission is accepted. Please make sure it is accurate and that any funding sources listed in your Funding Information later in the submission form are also declared in your Financial Disclosure statement.<br><br>View published research articles from *PLOS ONE* for specific examples.	The authors have declared that no competing interests exist.

**NO authors have competing interests**<br><br>Enter: *The authors have declared that no competing interests exist.*<br><br>**Authors with competing interests**<br><br>Enter competing interest details beginning with this statement:<br><br>*I have read the journal's policy and the authors of this manuscript have the following competing interests: [insert competing interests here]*<br><br>* typeset	
**Ethics Statement**<br><br>Enter an ethics statement for this submission. This statement is required if the study involved:<br><br>• Human participants<br>• Human specimens or tissue<br>• Vertebrate animals or cephalopods<br>• Vertebrate embryos or tissues<br>• Field research<br><br>Write "N/A" if the submission does not require an ethics statement.<br><br>General guidance is provided below. Consult the submission guidelines for detailed instructions. **Make sure that all information entered here is included in the Methods section of the manuscript.**	N/A

Powered by Editorial Manager® and ProduXion Manager® from Aries Systems Corporation

**Format for specific study types**<br><br>**Human Subject Research (involving human participants and/or tissue)**<br>• Give the name of the institutional review board or ethics committee that approved the study<br>• Include the approval number and/or a statement indicating approval of this research<br>• Indicate the form of consent obtained (written/oral) or the reason that consent was not obtained (e.g. the data were analyzed anonymously)<br><br>**Animal Research (involving vertebrate animals, embryos or tissues)**<br>• Provide the name of the Institutional Animal Care and Use Committee (IACUC) or other relevant ethics board that reviewed the study protocol, and indicate whether they approved this research or granted a formal waiver of ethical approval<br>• Include an approval number if one was obtained<br>• If the study involved *non-human primates*, add *additional details* about animal welfare and steps taken to ameliorate suffering<br>• If anesthesia, euthanasia, or any kind of animal sacrifice is part of the study, include briefly which substances and/or methods were applied<br><br>**Field Research**<br><br>Include the following details if this study involves the collection of plant, animal, or other materials from a natural setting:<br>• Field permit number<br>• Name of the institution or relevant body that granted permission	
**Data Availability**<br><br>Authors are required to make all data underlying the findings described fully available, without restriction, and from the time of publication. PLOS allows rare exceptions to address legal and ethical concerns. See the PLOS Data Policy and FAQ for detailed information.	Yes - all data are fully available without restriction

Powered by Editorial Manager® and ProduXion Manager® from Aries Systems Corporation

A Data Availability Statement describing where the data can be found is required at submission. Your answers to this question constitute the Data Availability Statement and **will be published in the article**, if accepted.<br><br>**Important:** Stating 'data available on request from the author' is not sufficient. If your data are only available upon request, select 'No' for the first question and explain your exceptional situation in the text box.<br><br>Do the authors confirm that all data underlying the findings described in their manuscript are fully available without restriction?	
**Describe where the data may be found in full sentences. If you are copying our sample text, replace any instances of XXX with the appropriate details.**<br><br>• If the data are **held or will be held in a public repository**, include URLs, accession numbers or DOIs. If this information will only be available after acceptance, indicate this by ticking the box below. For example: *All XXX files are available from the XXX database (accession number(s) XXX, XXX.*).<br>• If the data are all contained **within the manuscript and/or Supporting Information files**, enter the following: *All relevant data are within the manuscript and its Supporting Information files.*<br>• If neither of these applies but you are able to provide **details of access elsewhere**, with or without limitations, please do so. For example:<br><br>*Data cannot be shared publicly because of [XXX]. Data are available from the XXX Institutional Data Access / Ethics Committee (contact via XXX) for researchers who meet the criteria for access to confidential data.*<br><br>*The data underlying the results presented in the study are available from (include the name of the third party*	All relevant data are within the manuscript and its Supporting Information files.

and contact information or URL).<br>• This text is appropriate if the data are owned by a third party and authors do not have permission to share the data.<br><br>* typeset	
Additional data availability information:	

Powered by Editorial Manager® and ProduXion Manager® from Aries Systems Corporation

Manuscript Click here to access/download;Manuscript;Manuscript.docx

The role of export competitiveness in driving Renminbi cross-border settlement: Micro-level evidence from China

Danlu Bu[1], Lin Jiang[1,2*]

1. School of Accounting, Southwestern University of Finance and Economics, Chengdu, China,
2. School of Accounting, Sichuan Vocational College of Finance and Economics, Chengdu, China

*jianglin_90@163.com (LJ)

Author Contributions

Conceptualization: Danlu Bu
Data Curation: Lin Jiang
Investigation: Lin Jiang
Methodology: Danlu Bu, Lin Jiang
Project Administration: Danlu Bu
Software: Lin Jiang
Supervision: Danlu Bu
Writing – Original Draft Preparation: Lin Jiang
Writing – Review & Editing: Danlu Bu, Lin Jiang

Abstract

Cross-border trade settlement in Renminbi (RMB) is a crucial component of China's efforts to internationalize its currency. This study develops micro-level indicators of RMB cross-border settlement to empirically investigate the role of export competitiveness in promoting the use of RMB in international trade. The findings reveal that export competitiveness significantly enhances RMB cross-border settlement, with the establishment of Free Trade Zones further amplifying this effect. Heterogeneity analysis indicates that the impact is more pronounced in industries with higher concentration or in firms with greater operating liabilities. Moreover, the increased use of RMB in cross-border settlements, driven by export competitiveness, is associated with reduced import dependence and enhanced firm value. These results contribute to a deeper understanding of RMB internationalization through the lens of firm-level trade activities and offer valuable empirical insights for the strategic promotion of RMB internationalization.

1. Introduction

The internationalization of a nation's currency serves as a key indicator of its overall economic strength and plays a pivotal role in fostering international exchanges and cooperation. It enhances a country's global influence and strengthens its voice in international affairs, while also contributing to the stability and security of the national financial system. In today's globalized economy, currency internationalization has emerged as a major trend. As China's legal tender, the internationalization of RMB holds the potential to mitigate exchange rate risks and lower transaction costs in cross-border trade, thus facilitating international trade and investment. Moreover, RMB internationalization can stimulate growth in both China's macroeconomic and microeconomic sectors. Beyond economic benefits, it is also instrumental in safeguarding China's financial security and elevating its national credit and competitiveness on the global stage.

The internationalization of RMB can be defined as the process by which the RMB is able to cross national boundaries, circulate outside the country, and gradually become a widely recognized settlement, valuation and reserve currency in the international arena. RMB settlement refers to the agreement between two parties engaged in cross-border trade to use RMB as the settlement currency. For instance, when a Chinese enterprise exports goods to an overseas enterprise, it is agreed that the overseas enterprise will make payment in RMB. RMB valuation refers to the value of international commodities being calculated and labeled in RMB, with the prices of other national currencies being converted according to the exchange rate with RMB. It is also worth noting that the majority of international trade and financial transactions in the international market are now denominated in U.S.

dollars. Finally, the term RMB reserves refers to the fact that the RMB is used as the currency of foreign exchange reserves by countries around the world. In the first quarter of 2022, the share of RMB in global foreign exchange reserves reached 2.88 per cent, representing a 1.8 per cent increase compared with 2016, when RMB was first included in the Special Drawing Rights (SDR) basket of currencies. In terms of functionality, the valuation and reserve currency must be a currency that is widely used internationally for payment and settlement. This highlights the fundamental position of the RMB cross-border settlement function. Furthermore, international experience has demonstrated that currency internationalization can also entail the local currency acting solely as an international settlement currency. Consequently, RMB internationalization is also directly defined as a certain proportion of international trade transactions settled in RMB [1]. A review of the historical development of RMB internationalization in China reveals that the process of RMB internationalization is a gradual one, progressing from the initial stage of "settlement" to the subsequent stages of "valuation" and then to "reserve". In accordance with the principle of "resolving the easy before the difficult", it is recommended that the objective at this stage be the RMB settlement of cross-border trade [2], so to promote the internationalization of the RMB in an orderly manner.

As China's high-level opening up to the outside world continues to deepen, the proportion of RMB settlement in cross-border trade has been increasing, and is currently the most widely used major currency in the world. In 2023, the amount of RMB cross-border receipts and payments totaled RMB 52.3 trillion yuan, an increase of 24.2 per cent year-on-year. This has led to RMB becoming the first major settlement currency for China's cross-border receipts and payments. The total amount of cross-border RMB receipts and payments for trade in goods was RMB 10.7 trillion yuan, representing 25% of the total amount of cross-border receipts and payments for trade in goods in local and foreign currencies during the same period. This represents a 7 percentage point increase from 2022, indicating that cross-border trade settlement in RMB has made a certain degree of progress. Nevertheless, in comparison with international mainstream currencies such as the US dollar and the euro, the proportion of RMB settlement in cross-border trade remains relatively low. According to data released by the Society for Worldwide Interbank Financial Telecommunication (SWIFT), as of June 2023, the proportion of the US dollar in the global total settlement amount was 42.02%, ranking first, followed by the euro, the pound sterling and the Japanese yen with a proportion of 31.25%, 6.88% and 3.36%. The proportion of the RMB in the global total settlement amount is 2.77%, placing it in fifth place. This is a significant discrepancy from the currencies of the US dollar and the euro. The question of how to increase the ratio of RMB settlement in cross-border trade remains a key topic in the process of promoting the internationalization of the RMB in an orderly manner in China.

The theory of international trade settlement currency choice posits that competitiveness is the

primary factor in the contemporary international situation that determines which currency is selected for settlement in international trade. At the macro level, the choice of settlement currency reflects the advantages and disadvantages of a country's exchange rate system and the size of the spillover effect of its monetary policy on other countries. In addition, a country's political and military power [3], national credibility [4], and monetary policy stability [5], were all identified as crucial elements in the choice of currency for international trade settlements. This reflects the macro-guarantee of national competitiveness in a country's currency cross-border settlement choice. At the micro level, the bargaining power and economic strength of enterprises are of great significance for coping with the current complex trade environment and fighting for discourse advantages in international trade. This bargaining power may come from a variety of aspects, including product market share and product differentiation [6,7,8]. Concurrently, the capacity of firms to regulate costs represents a pivotal element in attaining an international competitive edge [9]. The international competitiveness of enterprises can amplify the domestic currency selection advantage, thereby enabling enterprises to occupy the currency initiative in cross-border trade settlement. This, in turn, gives rise to the micro-dynamics of a country's currency cross-border settlement choice. Consequently, enterprise export competitiveness represents a pivotal factor in the selection of an international trade settlement currency [10,11,12]. However, the majority of current studies on the relationship between enterprise export competitiveness and RMB cross-border settlement at the micro level are qualitative analyses, with few quantitative analyses due to the challenge of data sources. How is enterprise cross-border trade RMB settlement measured? Whether and how enterprise export competitiveness affects enterprise cross-border trade RMB settlement? And finally, how to promote RMB internationalization in an orderly manner? This paper seeks to address these questions.

This paper presents a micro-level measurement index of cross-border trade RMB settlement and empirically examines the impact of enterprise export competitiveness on cross-border trade RMB settlement. The results indicate that enterprise export competitiveness plays a significant role in facilitating enterprise cross-border trade RMB settlement. This conclusion remains consistent even after conducting a series of robustness tests, including replacing the measurement of the explanatory variables, lagging the period of the control variables, eliminating the influence of sample selection and excluding special samples. The mechanism test indicates that the establishment of China's Free Trade Zone (FTZ) can enhance the impact of enterprise export competitiveness on cross-border trade RMB settlement, which reflects the efficacy of the FTZ. Concurrently, the heterogeneity analysis indicates that the impact of enterprises' export competitiveness on cross-border trade settlement in RMB is more pronounced in the sub-sample with higher industry concentration or more operating liabilities. This highlights the pivotal role of industry-leading enterprises and the necessity for enterprises to reduce their reliance on financial liabilities. Furthermore, this paper reveals that the increase in the proportion of cross-border trade settlement in RMB due to enterprises' export

competitiveness can significantly diminish import dependence and increase the value of enterprises.

This paper makes several key contributions. First, it offers an innovative micro-level measurement of RMB cross-border trade settlement, shedding light on the current development of such settlements among enterprises. This contributes to both academic and practical understanding of RMB cross-border trade settlement and the broader process of RMB internationalization. Second, the study highlights the role of export competitiveness in driving RMB cross-border settlements. It provides valuable insights for enterprises to optimize resource allocation and improve the efficiency of factor integration, while offering practical guidance for policymakers to promote RMB internationalization in a structured and orderly manner. Third, the paper underscores the importance of FTZs in facilitating cross-border trade settlements, offering a perspective on the interaction between trade policies and currency internationalization, which can inform future policy design.

2. Theoretical analysis and hypothesis

In the early stages of the development of cross-border trade, the primary objective of the two parties involved in the trade was the cross-border purchase and sale of goods, with the subsequent flow of economic benefits being a significant factor. At this stage, the competition for global monetary sovereignty was not yet a significant concern, and thus, the convenience of the exporter's currency for cross-border trade settlements was a key consideration for both parties involved in the trade. This led to the theoretical study of the factors influencing the choice of international trade currencies, which began in the 1970s.

The theory of international trade settlement currency selection concerns the study of international trade and financial transactions in the settlement of countries, with a focus on the choice of currency as a medium of exchange. In 1973, the Swiss economist Grassman [13] conducted research on currency selection data from Sweden's import and export trade with Denmark in 1968 and 1973. His findings revealed that 66% of Sweden's merchandise exports were settled in Swedish currency, while only 25% were settled in Danish currency. Similarly, 59% of Sweden's merchandise imports were settled in Danish currency, while only 26% were settled in Swedish currency. Accordingly, the well-known rule of thumb that the choice of settlement and valuation currencies in international trade is based on the stickiness of commodities to national currencies, i.e., export commodities are mainly priced in exporters' currencies, is proposed. This reflects the important role of the subjective will of both trading parties in the choice of settlement currencies in cross-border trade at the early stage of the development of cross-border trade.

Nevertheless, as economic globalization deepens and more developing countries become involved in the division of labour within global value chains, the explanatory strength of the aforementioned rule is diminishing. The intensification of competition among countries has led to an

increase in the competition for monetary sovereignty. Consequently, enterprises in various countries have a preference for settling in their own currencies, whether for exports or imports. This has resulted in the capacity determinism of currency choice in cross-border trade. From a macroeconomic perspective, a country's military strength, economic level, and national credibility collectively constitute the source of a country's competitiveness. When an exporting country has a larger market share of commodities and stronger export competitiveness, its currency is more influential and acceptable in the international market. Consequently, there is a greater likelihood of adopting the exporting country's currency as the settlement currency [14,15]. A review of the internationalization of the world's major currencies reveals that the emergence of a national currency as the mainstream currency for global reserves and circulation has been supported by the country's comprehensive strength and international competitiveness. Prior to the eighteenth century, with the rise of the great powers of Portugal, Spain and the Netherlands, the gold and silver coins minted by these countries became the global circulation and reserve currency. From the early nineteenth century to the mid-twentieth century, Britain's naval dominance continued to strengthen, contributing to the pound sterling becoming the dominant paper currency at the time. The pound sterling was thus deservedly established as an international currency of equal status to gold. From that point onwards, it enjoyed a long period of monetary dominance, spanning 130 years. Following the outbreak of the Second World War, the United States reaped significant financial rewards through the sale of goods, emerging as the most successful nation in the conflict. Conversely, the United Kingdom suffered a significant setback during the war. With the United States assuming the role of the dominant global power, the US dollar gradually became the dominant currency in the international monetary system, a position it continues to occupy to this day. Other currencies, such as the Japanese yen and the Deutsche Mark, have also become important international currencies as a result of their respective economic strengths and international competitiveness.

At the micro level, Page [11] posits that an exporter's overseas market power can influence the choice of currency for trade settlement. This can result in reduced costs of settlement in the exporter's currency and avoidance of exchange rate risk. Magee [12] also indicates that the exporter's currency is relatively advantageous when the exporter has a greater competitive power in the negotiation of international trade contracts, thereby increasing the likelihood of international trade being settled in the exporter's currency. This suggests that export enterprises seeking to utilize the national currency as the cross-border trade settlement currency must demonstrate a certain degree of export competitiveness in order to overcome the other party's willingness to use their own national currency for the settlement of cross-border trade.

On the other hand, in global multilateral economic relations, developed country currencies such as the US dollar and the euro currently dominate cross-border trade [16], and even when non-main currency companies trade across borders, most tend to settle in third-party currencies [13,17,18],

reflecting inertia in the use of mainstream currencies [19]. And breaking out of this inertia is associated with higher transaction costs [20,21]. Currency substitution occurs on a large scale only when the new currency has a clear advantage [22]. Strong export competitiveness of firms, both in terms of resource advantage and capability advantage, reflects that firms are recognized by the international market, and for reasons such as the convenience of purchasing the firm's products, the need for long-term cooperation with the firm, and the promotion of consumption upgrading in the importing country, the importing firm shows a willingness to switch from the third country's currency to the exporting country's currency, and accordingly the currency of the country in which the exporting firm is located has the advantage of substituting for the dominant currency.

It can be seen that the competition for currency sovereignty has become an important object of competition among major countries, and national competitiveness has become the macro-guarantee for the cross-border settlement of a country's currency, while the export competitiveness of enterprises, which is based on the resource and capability advantages, has become the endogenous driving force for fighting for the currency initiative in international trade. Whether it is to increase the proportion of RMB settlement in bilateral trade relations or to break the settlement inertia of the dominant currency and switch to RMB settlement in the multilateral system, it is necessary for China's enterprises to improve their export competitiveness and enhance their discourse advantages.

Based on the preceding analysis, this paper posits the following hypothesis:

H1: Enterprise export competitiveness has a positive effect on promoting RMB cross-border settlement.

3. Methodology

The construction of RMB cross-border settlement indicators in this paper requires the use of foreign currency monetary items. However, the existence of undisclosed, incomplete disclosure and inconsistent format of foreign currency monetary items in the annual reports of listed companies before 2013 limits the collection of data for this paper. In 2014, the China Securities Regulatory Commission (CSRC) revised the "Information Disclosure and Reporting Rules for Companies with Publicly Issued Securities No. 15 - General Provisions on Financial Reporting", which coordinated information disclosure requirements and improved the degree of integration of information disclosure, and then the foreign currency monetary items in the annual reports of listed companies were standardized and comprehensively disclosed, which improved the accessibility of foreign currency data. Therefore, this paper selects all A-share listed companies from 2014 to 2022 as the initial sample, and conducts the following processing:(1) excluding companies with no overseas business income; (2) excluding the sample of listed companies in the financial industry; (3) excluding the sample of listed companies with incomplete data. The final sample of this paper is

2081 listed companies with a total of 10,026 observations for 9 years. The data of foreign currency monetary items for the calculation of RMB settlement of cross-border trade are manually extracted from the annual reports of enterprises, and the indicators for the calculation of other variables are obtained from the CSMAR database and the CNRDS database, and in order to maintain the stability of the model, all continuous variables are subjected to the upper and lower 1% shrinking tail treatment.

The dependent variable of this paper is RMB cross-border settlement. Based on the overseas sales and collection cycle of the enterprise, the overseas business revenue obtained by the enterprise goes into the money fund account when the payment is received, and goes into the accounts receivable account when the payment is not received.Therefore, this paper takes the sum of the increase in the amount of foreign currency settlement of RMB in the money fund and the increase in the amount of foreign currency settlement of RMB in the accounts receivable to the current year's overseas business revenue, i.e, the foreign currency settlement ratio (*FC*) , as a proxy indicator of RMB cross-border settlement. This indicator is a negative indicator, and a higher FC indicates a lower degree of RMB cross-border settlement.

The independent variable of this paper is export competitiveness (*COMP*). Liu et al. [23] proposed that enterprise resources and enterprise capabilities are two aspects to measure the international competitiveness of enterprises, and since then, scholars have mostly screened financial indicators and comprehensively evaluated the export competitiveness of enterprises from these two aspects. Based on the previous literature, this paper selects the following indicators and uses the entropy weighting method to weight and sum the indicators to reflect the export competitiveness of enterprises: (1) Total assets. Assets, as the most direct representative of firm resources, can intuitively reflect the resource-based export competitive advantage of firms; (2) the proportion of R&D personnel. Modern enterprise competition is ultimately talent competition, and human resources are the first resource of enterprises, which is the intrinsic foundation of enterprise competitiveness. General Secretary Xi pointed out that "the key to doing a good job in China is in the Party, the key is in the people, and the key is in the talents". Therefore, this paper takes the ratio of the number of R&D personnel to the total number of personnel as one of the indicators reflecting the resource-based export competitiveness of enterprises; (3) Asset-liability ratio. Financing constraint is the key to constraining the development of enterprises, and the competitiveness of enterprises in the international arena largely depends on whether they can finance enough funds to support international operation and development. Therefore, this paper uses the asset-liability ratio to measure the financing capacity of enterprises as one of the indicators reflecting the capability-based export competitiveness of enterprises; (4) Profitability. Strong profitability is the basis for the survival of the enterprise, but also the key for the enterprise in the international competition to get a head start, so this paper takes the profit margin as one of the indicators to reflect the capability-based

export competitiveness of enterprises; (5) the growth rate of operating income. Revenue growth is the best reflection of the continuous development and expansion of enterprises, so this paper takes the growth rate of operating income to measure the development ability of enterprises, as one of the indicators to reflect the export competitiveness of enterprise capacity; (6) Market share. How much market share the enterprise occupies is an important performance of competitiveness, which can reflect whether the enterprise has strong market influence and competitiveness in the industry. This paper adopts the proportion of a single enterprise's operating income to the total operating income of all enterprises in the same industry to measure enterprise market share as one of the indicators reflecting the capability-based export competitiveness of enterprises; (7) Cost advantage. The establishment of an enterprise's cost advantage is inextricably linked to its export competitiveness. Cost management plays an important role in enterprise development, and with advanced cost management capabilities, enterprises can gain competitive advantages in increasingly complex international trade [9]. Based on Poter [9], this paper uses the ratio of operating income to operating costs as a proxy variable for cost advantage as one of the indicators reflecting the capability-based export competitiveness of enterprises. Considering that the impact of firms' export competitiveness on RMB cross-border settlement may have a time lag, and also to avoid the endogenous effect of reverse causality, this paper substitutes export competitiveness lagged by one period as an independent variable in the model.

In addition, this paper selects firm age (*AGE*), accounts receivable turnover (*ART*), capital intensity (*KL*), return on assets (*ROA*), tradable shares ratio (*TS*), cash flow ratio (*CF*) and type of ownership (SOE) as control variables to control for other aspects of firm characteristics, and the specific meanings of each variable are shown in S1 Appendix. To prevent the omission of other firm-level influences and yearly macroeconomic influences, this paper constructs a panel data model as in equation (1) and controls for individual (*ID*) and year (*YEAR*) double fixed effects, and expects the coefficient of *COMP* to be significantly negative if the hypothesis holds.

$$FC_{it} = \alpha + \beta \cdot COMP_{i,t-1} + \sum controls_{it} \cdot \gamma + YEAR_t + ID_i + \varepsilon_{it} \quad (1)$$

4. Empirical results

4.1 Summary statistics

Table 1 shows the descriptive statistics of the variables. It can be seen that in the full sample, the mean value of FC is 0.025, the minimum value is -5.691, and the maximum value is 3.968, which indicates that the proportion of foreign currency settlements of listed companies has a relatively even distribution, and the average proportion of foreign currency settlements at the firm-year level is 2.5 per cent. In addition, the mean value of export competitiveness (*COMP*) is 0.21, the minimum value

is 0.167 and the maximum value is 0.303, which shows that there are some differences in export competitiveness among companies. Corresponding descriptive statistics were also carried out for other control variables.

Table 1. Summary statistics

Variables	Observations	Mean	Std	Min	Max
FC	10026	0.025	0.903	-5.691	3.968
COMP	10026	0.210	0.025	0.167	0.303
AGE	10026	2.951	0.283	2.197	3.638
KL	10026	2.227	1.420	0.451	9.231
TS	10026	0.771	0.218	0.247	1
ROA	10026	0.029	0.085	-0.414	0.204
ART	10026	2.488	1.709	0.905	12.258
CF	10026	0.050	0.064	-0.132	0.236
SOE	10026	0.209	0.407	0	1

Fig 1 shows the annual trend of the proportion of foreign currency settlement of enterprises. It can be seen that from 2014 to 2022, the proportion of foreign currency settlement of enterprises shows a gradual downward trend. In 2014, RMB cross-border settlement was at the stage of initiation and rapid development, and about 25% of China's cross-border trade was settled in RMB, so the proportion of Chinese enterprises' foreign currency settlement was lower in 2014. On 11 August 2015, China implemented the RMB exchange rate reform, which revoked the policy of keeping the RMB stable against the US dollar and replaced it with an exchange rate adjustment with reference to a basket of currencies. Influenced by the market volatility caused by the reform, the subsequent depreciation of the RMB and the slowdown in capital account opening, as well as the trade friction between China and the US in 2018, the development of RMB cross-border settlement in China was in a relatively stagnant stage from 2015 to 2018, resulting in a relatively low proportion of foreign currency settlement from 2015 to 2018, as shown in Fig 1. After 2019, China accelerated the pace of financial opening, strongly promoted the international use of RMB by signing and updating the RMB bilateral currency swap agreement, and cooperated with the Bank for International Settlements to set up the RMB liquidity arrangement and other measures, and the company's cross-border trade gradually expanded the proportion of RMB settlement, so that RMB cross-border settlement ushered in a rapid development period. Fig 1 shows that in 2019, the proportion of foreign currency settlement was -5.05%, which was the first time that the foreign currency settlement achieved negative growth. From 2020 to 2022, the proportion of foreign currency settlement is at a low level, indicating that the momentum of RMB cross-border settlement by enterprises in China is favourable.

Fig 1. The annual trend in average foreign currency settlement share

The annual trend of RMB cross-border settlement by enterprises reflected in this micro-level

data is in line with the stage-by-stage macro direction of the RMB internationalization process in China, indicating that RMB cross-border settlement is truly the foundation and the first step in promoting RMB internationalization, and also reflects the feasibility and validity of the measurement of RMB cross-border settlement by enterprises constructed in this paper, which provides the data basis for the research.

4.2 Regression analysis

The basic regression results of export competitiveness and foreign currency settlement share are shown in Table 2. Columns (1)-(3) are the regression results of uni-variate regression, regression controlling only year and individual fixed effects, and regression adding all control variables, respectively, which show that export competitiveness is significantly negatively correlated with foreign currency settlement share, and all of them are valid at the 5% significance level. And the coefficient of *COMP* in column (3) shows that for each unit increase in the export competitiveness of the firm, the share of foreign currency settlement is reduced by 214.2%, which means that the export competitiveness of the firm can significantly promote RMB cross-border settlement. It supports the hypothesis of this paper.

Table 2. The impact of export competitiveness on RMB cross-border settlement

	FC		
	(1)	(2)	(3)
COMP	-1.144**	-2.130**	-2.142**
	(-2.50)	(-2.26)	(-2.29)
AGE			-0.192
			(-0.72)
KL			-0.015
			(-0.59)
TS			-0.123
			(-1.49)
ROA			0.917***
			(4.58)
ART			-0.036
			(-1.59)
CF			0.136
			(0.60)
SOE			-0.338***
			(-3.77)
Year FE	no	yes	yes
Firm FE	no	yes	yes
N	10026	10026	10026
R^2	0.002	0.015	0.025

Robust standard error adjusted t-values are shown in brackets. *, **, *** denote 10%, 5% and 1% levels of significance respectively.

4.3 Robustness test

4.3.1 Replacement of explanatory variable measures

Based on the overseas sales and collection cycle, this paper designed a measure of RMB cross-border settlement by the ratio of the increase in foreign currency accounts receivable and foreign currency monetary funds to overseas business revenue, which is reasonable and feasible. However, considering that part of the increase in foreign currency monetary funds may come from the separate foreign currency reserves of enterprises rather than overseas business income, this paper measures the degree of RMB cross-border settlement as the ratio of the increase in foreign currency accounts receivable only to overseas business income (*FC_new*) and re-runs the regression.

The results are shown in column (1) of Table 3, and the coefficient of *COMP* is still significantly negative, confirming that export competitiveness can indeed promote RMB cross-border settlement.

Table 3. Robustness test

	FC_new	*FC*	*SELECT*	*FC*	*FC*
	(1)	(2)	(3)	(4)	(5)
COMP	-0.589**	-1.695*		-2.190**	-2.247*
	(-1.97)	(-1.76)		(-2.34)	(-1.91)
AGE	-0.101	-0.199	0.098***	0.038	-0.260
	(-1.12)	(-0.83)	(3.69)	(0.33)	(-0.81)
KL	-0.016**	-0.021	-0.095***	0.006	-0.014
	(-2.33)	(-1.09)	(-21.36)	(0.21)	(-0.50)
TS	-0.055**	-0.131*	0.586***	-0.197*	-0.120
	(-2.32)	(-1.78)	(18.99)	(-1.75)	(-1.28)
ROA	0.272***	0.385**	-0.331***	0.990***	1.038***
	(4.07)	(2.16)	(-3.08)	(4.66)	(4.54)
ART	-0.025***	-0.021	-0.067***	-0.005	-0.031
	(-3.70)	(-1.04)	(-16.68)	(-0.37)	(-1.07)
CF	-0.223***	0.268	0.693***	0.026	0.021
	(-2.89)	(1.26)	(5.80)	(0.10)	(0.09)
SOE	-0.056**	-0.344***	-0.436***	-0.289***	-0.344***
	(-2.19)	(-3.83)	(-23.40)	(-2.76)	(-3.59)
INV			-0.014***		
			(-17.46)		
IMR				-0.154	
				(-0.90)	
Year FE	yes	yes	yes	yes	yes
Firm FE	yes	yes	yes	yes	yes
N	10026	10026	32767	10026	8683
R^2	0.031	0.021	0.109	0.025	0.025

Robust standard error adjusted t-values are shown in brackets. *, **, *** denote 10%, 5% and 1% levels of significance respectively. Each

control variable in column (2) is lagged by one period. Column (3) has observations of 32,767 due to the inclusion of other unselected data using a probit model, so the R^2 there is reported as a pseudo R^2. In column (5) some samples are excluded, so the observations becomes 8,683.

4.3.2 Control variables lagged by one period

To mitigate the possible problem of reverse causation between the control variables and the dependent variable, the paper lags all other control variables by one period and re-runs the regression.

The regression results are shown in column (2) of Table 3, where the coefficient on *COMP* is negative and holds at the 10% significance level. It can be seen that after accounting for the time lag of all variables, the export competitiveness of firms still significantly promotes RMB cross-border settlement, indicating that the conclusion of this paper is robust.

4.3.3 Adjustment for the effect of sample selection

The measurement of RMB cross-border settlement indicators requires the use of overseas business revenue, so the samples in this paper are all enterprises with overseas business revenue, which may cause the results of this paper to contain selectivity bias. In order to eliminate the influence of sample selection problems, this paper applies the two-stage Heckman method to correct the selectivity bias.

Column (3) in Table 3 is the result of the first stage, i.e., using the probit model to estimate the probability of enterprises generating overseas business income (*SELECT*) with all the control variables in this paper plus the amount of enterprises' overseas investment (*INV*). Column (4) is the result of the second stage, which is obtained by calculating the Inverse Mills Ratio (*IMR*) with the estimated value of the regression result from the first stage and adding it to the original model as a control variable. It can be seen that after selective bias adjustment, the export competitiveness of enterprises still has a significant promoting effect on RMB cross-border settlement.

4.3.4 Special sample exclusion

In 2015, China implemented the RMB exchange rate reform, reversing the policy of keeping the RMB stable against the US dollar and allowing the exchange rate to float freely within a certain range. In the same year, the RMB joined the International Monetary Fund's Special Drawing Rights (SDR) currency basket, becoming an emerging market currency. As a result, the volume of RMB cross-border settlement increased significantly in 2015. Moreover, in 2018, the trade friction between the United States and China emerged, and the United States raised tariffs on certain goods in China, which had a greater impact on some industries, leading to an abnormal trend in RMB cross-border settlement.

Therefore, to exclude the impact of the special event in 2015 and specific industries in 2018,

this paper re-runs the regressions excluding the entire sample in 2015 and some industry samples in 2018. The results in column (5) of Table 3 show that the conclusion of this paper is robust.

4.4 Mechanism

In order to overcome the disadvantages of multinational enterprises in international business, the support of the home country government is of great importance, which can help enterprises turn disadvantages into advantages and improve international competitiveness [24]. Free trade zones (FTZs) are an important fulcrum for China to develop a high degree of openness to the outside world, playing an early and pilot role in promoting the liberalization and facilitation of foreign trade, as well as an important practice of the Chinese government to support export enterprises. By 2022, China has established FTZs in 21 provinces and municipalities, including Shanghai, Guangdong, etc., which provide enterprises with greater advantages and convenience in conducting export business.

Therefore, this paper identifies whether enterprises are located in the region where the FTZs is established, and constructs the cross-multiplier of the dummy variable for the establishment of the FTZs (*ZONE*) and the export competitiveness (*COMP*ZONE*), which is added to the original model to re-run the regression. The results are shown in column (1) of Table 4, and it can be seen that the coefficient of the cross-multiplier term is significantly negative, indicating that the establishment of FTZs strengthens the promoting role of export competitiveness on RMB cross-border settlement.

Table 4. Mechanistic and heterogeneity analyses

	FC	*FC* High industry concentration	*FC* Low industry concentration	*FC* Financial liability	*FC* Operating liability
	(1)	(2)	(3)	(4)	(5)
COMP	-0.827	-4.862**	-0.091	0.571	-3.972***
	(-0.71)	(-2.37)	(-0.07)	(0.39)	(-2.86)
ZONE	0.467**				
	(2.43)				
*COMP*ZONE*	-2.040**				
	(-2.16)				
AGE	-0.202	-0.799	-0.656	-0.464	0.082
	(-0.76)	(-1.30)	(-1.39)	(-1.16)	(0.20)
KL	-0.014	-0.081*	0.056	-0.039	-0.022
	(-0.57)	(-1.90)	(1.56)	(-1.24)	(-0.53)
TS	-0.117	-0.177	-0.035	-0.096	-0.115
	(-1.42)	(-1.20)	(-0.26)	(-0.82)	(-0.87)
ROA	0.909***	0.938***	0.838***	0.799***	1.112***
	(4.55)	(2.86)	(3.63)	(2.98)	(3.37)
ART	-0.036	-0.047	-0.006	-0.083***	-0.005
	(-1.58)	(-0.86)	(-0.21)	(-2.79)	(-0.12)
CF	0.140	0.217	-0.242	0.443	-0.048
	(0.62)	(0.64)	(-0.68)	(1.32)	(-0.15)

SOE	-0.340***	-0.410**	-0.278*	-0.241***	-0.344*
	(-3.79)	(-2.18)	(-1.79)	(-2.72)	(-1.85)
Year FE	yes	yes	yes	yes	yes
Firm FE	yes	yes	yes	yes	yes
N	10026	5013	5013	5013	5013
R^2	0.026	0.029	0.024	0.024	0.033
Diff		-4.771*		-4.543*	
p-value		(0.080)		(0.060)	

P-values are shown in the brackets of differences in coefficients between groups, and robust standard error adjusted t-values are shown in other brackets. *, **, *** denote 10%, 5% and 1% levels of significance respectively.

4.5 Heterogeneity analysis

4.5.1 Heterogeneity of industry concentration

The degree of industry concentration reflects the extent to which the leading enterprises in the industry control the market, and is a measure of the market structure of the industry, reflecting the degree of competition and monopoly in the market. A high degree of industry concentration means that there are several large enterprises in the industry that lead the development of the whole industry, and they may be able to play a pioneering role in export competitiveness and RMB cross-border settlement. Therefore, this paper adopts the industry Lerner index to measure industry concentration, and divides the sample into two groups of high and low industry concentration according to whether the index is higher than the median, and conducts group regression.

The regression results are shown in columns (2)-(3) of Table 4. The coefficient of COMP is significantly negative in the high industry concentration group, while it is not significant in the low industry concentration group, and the coefficient difference test between the groups shows that the coefficients of the two groups are significantly different, indicating that export competitiveness plays a stronger role in promoting RMB cross-border settlement for the enterprises with higher industry concentration. This reflects the spillover effect of the industry leaders in driving the scale of RMB cross-border settlement in the whole industry.

4.5.2 Heterogeneity of liability structure

The liabilities of an enterprise can be classified according to their purpose and source into financial liabilities, which refer to borrowings from financial institutions, the issue of bonds or other forms of debt, usually requiring the payment of high interest rates, and operating liabilities, which refer to liabilities associated with the day-to-day operations of the enterprise, including trade credit arising from the purchase of raw materials, the acquisition of machinery and equipment, and so on. A company's liability structure reflects its propensity for endogenous or exogenous financing. If a company's financial liabilities are much higher than its operating liabilities, this may indicate that the company is over-reliant on borrowed capital and that there is greater pressure on operating cash flow.

At the same time, higher operating liabilities, which are mainly commercial credit, also reflect a firm's higher market position in the industry [25]. Therefore, in this paper, the liability structure of firms is measured by the ratio of financial liabilities to total liabilities, and the group regression is conducted by dividing the sample into two groups, financial liability type and operating liability type, according to whether the ratio of financial liabilities is higher than the median.

The results are reported in columns (4)-(5) of Table 4. The coefficient of *COMP* is significantly negative in the operating liability type enterprises, but insignificant in the financial liability type enterprises, and passes the test of difference in coefficients between groups. The results indicate that the promoting effect of export competitiveness on RMB cross-border settlement is more significant in the enterprises with more operating liabilities, reflecting the importance of enterprises reducing the dependence on financial liabilities and accumulating the operating cash flow.

5. Further analyses

5.1 Further analysis of import dependence

In the current global production network, the division of labour among suppliers at all levels is highly specialized, which brings efficiency to the deep integration of global trade, but also increases the degree of import dependence [26]. For example, China's manufacturing have a high degree of import dependence in high-end parts and components, which is an obvious competitive disadvantage. Agriculture is even more so, as a high degree of import dependence increases the risk of food shortages caused by external shocks [27].

RMB cross-border settlement reflects the competitiveness of enterprises in the international market, and this competitiveness should be able to enhance the initiative and import options in foreign trade and reduce the supply chain risks caused by import dependence. Due to the limitations of import data collection, this paper measures the import dependence of enterprises by overseas business cost (*DEPEND*) and takes it as the dependent variable, and conducts a linear regression with the cross-multiplier term of export competitiveness and the inverse of foreign currency settlement share (*COMP*FC_r*) as the independent variable. The regression results are shown in column (1) of Table 5, and the coefficient of the cross-multiplier term is significantly negative, indicating that after RMB cross-border settlement promoted by export competitiveness, enterprises reduce their import dependence and enhance their risk coping ability and supply chain resilience.

Table 5. Further analyses

	DEPEND	*TobinQ*
	(1)	(2)
*COMP*FC_r*	-0.835***	0.078*
	(-3.18)	(1.89)

AGE	5.678**	-0.098
	(2.47)	(-0.19)
KL	-0.246***	-0.110***
	(-2.64)	(-5.74)
TS	-0.044	1.536***
	(-0.08)	(15.33)
ROA	-1.454*	0.927***
	(-1.71)	(5.59)
ART	-0.062	0.003
	(-0.59)	(0.12)
CF	0.313	1.055***
	(0.28)	(5.42)
SOE	-0.174	-0.109*
	(-0.35)	(-1.83)
Year FE	yes	yes
Firm FE	yes	yes
N	10026	9873
R^2	0.016	0.332

Robust standard error adjusted t-values are shown in brackets. *, **, *** denote 10%, 5% and 1% levels of significance respectively. There are 153 missing values for the TobinQ in column (2), so the observations are reduced to 9873.

5.2 Further analysis of firm value

In the capital market, investors' confidence and investment decisions in a listed company can ultimately be reflected in the value of the company through changes in the share price. Strong export competitiveness and the resulting increase in the level of RMB cross-border settlement means that the company is also competitive in the capital market and may have higher future earnings, so investors are optimistic about the company's stock and push up the firm value.

In this paper, firms' TobinQ value (*TobinQ*) is used to characterize the firm value as the dependent variable, and the cross-multiplier term (*COMP*FC_r*) is used as the independent variable to conduct a linear regression to further explore the impact of export competitiveness and RMB cross-border settlement on the firm value. From the results in column (2) of Table 5, it can be seen that the coefficient of the cross-multiplier term is significantly positive, indicating that the increase in the degree of RMB cross-border settlement under the influence of export competitiveness can significantly increase the firm value, which facilitates enterprises' access to capital market support, promotes the development of the real economy and creates a virtuous circle.

6. Conclusions

Based on the micro measurement of the degree of RMB cross-border settlement, this paper examines the impact of enterprises' export competitiveness on RMB cross-border settlement. The empirical results show that the higher the export competitiveness of enterprises, the higher the degree of RMB

cross-border settlement. After a series of robustness tests of substituting the measurement of the dependent variable, lagging the period of the control variables, excluding the endogeneity effect of sample selection, and excluding the special samples, the facilitating effect of enterprises' export competitiveness on RMB cross-border settlement is confirmed.

Through the mechanism analysis, this paper finds that the construction of FTZ can significantly strengthen the promotion of RMB cross-border settlement by enterprises' export competitiveness, which highlights the role of government policies in the process of enterprise development; the heterogeneity analyses show that the impact of enterprises' export competitiveness on RMB cross-border settlement is more significant in the sub-sample of industry with higher concentration or debt structure with more operating liabilities, which highlights the importance of leading enterprises to drive the development of the whole industry and the importance of enterprises to reduce borrowing dependence and enhance operational flexibility. This paper also finds that improving RMB cross-border settlement under the influence of enterprises' export competitiveness can: (1) reduce import dependence and improve their risk management capability and supply chain resilience. (2) Enhance corporate value and promote the interactive cycle of the capital market and the real economy.

Based on the conclusions drawn from this study, the following suggestions are proposed. First, enterprises should focus on optimizing resource allocation to enhance their operational, financial, and developmental capabilities. This strategic improvement will enable them to secure competitive advantages in the international market, thereby promoting RMB cross-border settlement. Additionally, firms should aim to restructure their liabilities by reducing dependence on external borrowing so as to enhance operational flexibility. Second, industry-leading enterprises should assume a pivotal role in disseminating their competitive advantages throughout the sector. By fostering collaboration and knowledge-sharing, these enterprises can enhance the overall competitiveness of their industry, which in turn will contribute to higher levels of RMB cross-border settlement. Third, in the pursuit of orderly RMB internationalization, it is imperative for the government to bolster policy support for export-oriented businesses. Encouraging more enterprises to engage in foreign trade through the establishment of FTZs will facilitate trade liberalization and ease transactional barriers. Furthermore, priority should be given to enhancing policy incentives for leading firms, thereby maximizing the spillover effects of their competitive advantages across the industry.

References

[1] Gagnon JE, Troutman K. Internationalization of the renminbi: The role of trade settlement. Policy briefs. 2014.

[2] Sun J, RMB for cross-border settlement or RMB internationalization? Studies of International Finance. 2014; 4:39-49.

[3] Cohen BJ. The future of sterling as an international currency. The Economic Journal. 1972; 82(326): 748-9. https://doi.org/10.2307/2230031.

[4] Dobson W, Masson PR. Will the renminbi become a world currency? China Economic Review. 2009; 20(1): 124-35. https://doi.org/10.1016/j.chieco.2008.05.005.

[5] Devereux MB, Engel C. Endogenous currency of price setting in a dynamic open economy model. NBER Working Paper. 2001; No. w8559. https://doi.org/10.3386/w8559.

[6] Feinberg RM. The interaction of foreign exchange and market power effects on German domestic prices. The Journal of Industrial Economics. 1986; 35(1): 61-70. https://doi.org/10.2307/2098606.

[7] Feenstra RC, Gagnon JE, Knetter MM. Market share and exchange rate pass through in world automobile trade. Journal of International Economics. 1996; 40(1-2): 187-207. https://doi.org/10.1016/0022-1996(95)01402-0.

[8] McKinnon RI. Optimum currency areas. The American Economic Review. 1963; 53(4): 717-25.

[9] Porter ME. Competitive advantage: Creating and sustaining superior performance. New York: The Free Press; 1985.

[10] Viaene JM, de Vries CG. International trade and exchange rate volatility. European Economic Review. 1992; 36(6): 1311-21. https://doi.org/10.1016/0014-2921(92)90035-U.

[11] Page SAB. The choice of invoicing currency in merchandise trade. National Institute Economic Review. 1981; 98: 60-72. https://doi.org/10.1177/002795018109800105.

[12] Magee SP. Currency contracts, pass through, and devaluation. Brookings Papers on Economic Activity. 1973; 4(1): 303-25.

[13] Grassman SA. Fundamental symmetry in international payment patterns. Journal of International Economics. 1973; 3(2): 105-16. https://doi.org/10.1016/0022-1996(73)90035-4.

[14] Bacchetta P, van Wincoop E. A theory of the currency denomination of international trade. Journal of International Economics. 2005; 67(2): 295-319. https://doi.org/10.1016/j.jinteco.2004.01.006.

[15] Fukuda S, Ono M. On the determinants of exporters' currency pricing: History vs. expectations. Journal of the Japanese and International Economies. 2006; 20(4): 548-68. https://doi.org/10.1016/j.jjie.2006.07.003.

[16] Magee SP, Rao RK. Vehicle and nonvehicle currencies in international trade. The American Economic Review. 1980; 70(2): 368-73.

[17] Tavlas GS. The international use of the US dollar: An optimum currency area perspective. World Economy. 1997; 20(6): 709-47. DOI:10.1111/1467-9701.00098.

[18] Gopinath G. The international price system. A report from National Bureau of Economic Research. 2015.

[19] Krugman P. Scale economies, product differentiation, and the pattern of trade. The American Economic Review. 1980; 70(5): 950-9.

[20] Rey H. International trade and currency exchange. The Review of Economic Studies. 2001; 68(2): 443-64. https://doi.org/10.1111/1467-937X.00176.

[21] Witte MD. The currency denomination of external European Union imports after European Union expansion. Economic Systems. 2009; 33(3): 245-58. https://doi.org/10.1016/j.ecosys.2009.05.003.

[22] Dowd K, Greenaway D. Currency competition, network externalities and switching costs: Towards an alternative view of optimum currency areas. The Economic Journal. 1993; 103(420): 1180-9. https://doi.org/10.2307/2234244.

[23] Liu W, Zhang Q, Xie J. Empirical research on international competitiveness of central enterprises. Chinese Public Administration. 2009; 8: 121-3.

[24] Prasannath V, Adhikari RP, Gronum S, Miles PM. Impact of government support policies on entrepreneurial orientation and SME performance. International Entrepreneurship and Management Journal. 2024; https://doi.org/10.1007/s11365-024-00993-3.

[25] Fisman R, Raturi M. Does competition encourage credit provision? Evidence from African trade credit relationships. Review of Economics and Statistics. 2004; 86(1): 345-52. https://doi.org/10.1162/003465304323023859.

[26] Gereffi G, Humphrey J, Sturgeon T. The governance of global value chains. Review of International Political Economy. 2005; 12(1):78-104. https://doi.org/10.1080/09692290500049805.

[27] Huang J, Yang G. Understanding recent challenges and new food policy in China. Global Food Security.2017; 12(3):119-26.https://doi.org/10.1016/j.gfs.2016.10.002.

Supporting information

S1 Appendix. Variables definition.

S2 Fig. The annual trend in average foreign currency settlement share.

S3 Appendix. Data.

Appendix

Click here to access/download
Supporting Information
S1_Appendix.docx

Fig

Click here to access/download
Supporting Information
S2_Fig.docx

Click here to access/download
Supporting Information
S3_Appendix.xlsx
