# Supplementary material for: The role of export competitiveness in driving Renminbi cross-border settlement: Micro-level evidence from China
Source: PLoS One. 2025 Apr 11;20(4):e0318099. doi: 10.1371/journal.pone.0318099 (PMC11990576; doi:10.1371/journal.pone.0318099)
Supplement: Attachment — Submitted filename: Response to Reviewers.docx [file pone.0318099.s004.docx]

**RE: Manuscript “The role of export competitiveness in driving Renminbi cross-border settlement: Micro-level evidence from China”**

Dear Reviewers,

We would like to sincerely thank you for the valuable comments and constructive feedback on our manuscript. We have carefully considered each point raised and have made the necessary revisions to improve the quality of the paper. Below, we provide a detailed response to each of the reviewers’ comments.

- **Reviewer #1:**

**Comment 1:**

**The idea and objective is important. The sample reached is significantly important. However, the panel analysis of the companies lacks proper formation and interpretation falls short of expectations. The general economic view of internationalization and importance of economic gain for exports in national currency was used without proper scientific support. Therefore results and suggestions are also too general.**

**To emphasize again, the objective and processing is important especially for china but the analytical process and inferences need to be over viewed. The exchange rates of significant trade partners' or at lease USD itself could be considered as an explanatory.**

**Re:** Thank you for this valuable comment. In response, we have revisited the methodology section to refine the model specifications and ensure that the interpretation of the coefficients is more precise and aligned with theoretical expectations. Specifically, we have taken your suggestion regarding the inclusion of exchange rates into account, and added three additional control variables: exchange rate (ER), government subsidy ratio (SUB), and the proportion of overseas business income (OI). This adjustment helps to invite important external factors that may influence cross-border RMB settlement into the model and improve the robustness and credibility of the results. The inclusion of these control variables is discussed in detail in the revised manuscript, and the regression results are presented in the updated tables.

In addition, we have supplemented our theoretical framework by incorporating additional references to relevant literature on the internationalization of currencies and the role of it in the world monetary system. We now provide a more solid theoretical basis for linking export competitiveness with RMB cross-border settlement and highlight the economic logic behind them. Specifically, in *Introduction* section, we have incorporated the comparative case of Konka and Zoomlion to elucidate why micro-level competitiveness can influence RMB cross-border settlement beyond macro-level factors. And in *Theoretical analysis and hypothesis* section, we have strengthened the theoretical support by including a more thorough discussion from both theoretical and practical perspectives, with the case of Media and ZTO helping to further explain the possibility that companies improving their export competitiveness may have more RMB cross-border settlement share. In this part, an “argument vs. counter-arguments interactions” method has been used in the revised version to address the research inquiries in a systematic and effective manner. This revision should offer a stronger scientific foundation for the arguments presented in the paper.

**Comment 2:**

The reviewer made several comments in the form of in-text annotations, and we respond to each annotation individually as follows.

**1. Some sentences lack evidence or scientific proof.**

**Re**: We have added references to those sentences or revised our expression.

“It bolsters a country’s global influence and its role in the world monetary system, while also contributes to the stability and security of international trade [1]” ([1] Cohen BJ. Currency power: Understanding monetary rivalry. Princeton University Press; 2018.)

“At this stage, the competition for global monetary sovereignty was not yet a prominent concern, and thus, the convenience of the exporter’s currency for cross-border trade settlements became a key consideration for both parties involved in the transaction [11]” ([11] Grassman SA. Fundamental symmetry in international payment patterns. Journal of International Economics. 1973; 3(2): 105-16. [https://doi.org/10.1016/0022-1996(73)90035-4](https://doi.org/10.1016/0022-1996(73)90035-4).)

**2. “export commodities are mainly priced in exporters' currencies, is proposed”, this is an a priori knowledge but can be examplified quantitatively.**

**Re**: In the revised manuscript, we used Grassman’s finding to illustrate this knowledge, and adjusted the words to “His findings revealed that 66% of Sweden’s merchandise exports were settled in Swedish currency, while only 25% were settled in Danish currency. Similarly, 59% of Sweden’s merchandise imports were settled in Danish currency, while only 26% were settled in Swedish currency. This indicates that export commodities are mainly priced and settled in the exporters’ currencies. Grassman’s findings led to the formulation of the well-known rule of thumb: the choice of settlement and valuation currencies in international trade is based on the stickiness of commodities to national currencies.”

**3. USA is also a major importer in many sectors and its main partner is china... the potential earnings of both parties can be exemplified here or this is expected in the later stages of the paper.**

**Re**: In the revised manuscript, we have added a discussion that exemplified the economic benefits for USA as an importer and for China as an emerging economy. The words are as follows:

“Following World War II, the United States, having emerged as the war’s largest beneficiary, gradually replaced the UK as the world’s leading power. The US dollar then rose to dominance in the international monetary system. Despite being a major importer, the United States’ extensive bilateral trade conducted in dollars further cemented the dollar’s global position. Similarly, the Japanese yen and Deutsche Mark became important international currencies due to their respective economies’ strength and competitiveness. In the 21st century, China gradually emerged as the world’s second-largest economy, with its overall national power continuing to grow. While the RMB still lags behind currencies like the US dollar, its share in global cross-border settlements has steadily increased, demonstrating that the enhancement of national competitiveness has provided strong support for the internationalization of the RMB.”

**4. The R^2^ is too low. where is f statistic and its interpretation?**

**Re**: Thanks for the insightful comments. We appreciate your concerns regarding the low R² value in the model. We would like to provide some clarifications to address this issue and reinforce the validity of our findings.

Firstly, while it is true that the choice of cross-border settlement currency is influenced by many external factors, the significant statistical and economic impact of export competitiveness on RMB cross-border settlement in our model strongly supports the reliability of the findings. This highlights the importance of micro-level factors in explaining the decisions of firms regarding currency choice, particularly in the context of RMB internationalization.

Secondly, we have included three additional control variables—exchange rate (ER), government subsidy to revenue ratio (SUB), and overseas income ratio (OI)—to account for relevant macroeconomic and external factors. These variables have helped to improve the model's fit and provide a more comprehensive analysis of the issue.

Finally, we have reported the F-statistic for the model, which confirms the overall validity of the regression and ensures that the model is statistically sound.

**5. “This paper also finds that improving RMB cross-border settlement under the influence of enterprises' export competitiveness can: (1) reduce import dependence and improve their risk management capability and supply chain resilience. (2) Enhance corporate value and promote the interactive cycle of the capital market and the real economy.” This is general evaluation. no case specific inference.**

**Re**: Thank you for your thoughtful and constructive feedback. We have revised the conclusion section to incorporate more specific inferences as follows:

“Based on the micro measurement of the degree of RMB cross-border settlement, this paper examines the impact of enterprises’ export competitiveness on RMB cross-border settlement. The results show that stronger export competitiveness significantly promotes the use of RMB in cross-border trade, highlighting the critical role of firm-level strength as a micro-level factor in gaining currency autonomy in bilateral trade. Robustness tests, including changes in the measurement of explanatory variables, lagging control variables, and addressing sample selection biases, further confirm the generalizability and reliability of this effect.

Mechanism analysis reveals that the development of FTZs, through improved business environments and policy support, significantly enhances the effect of export competitiveness on RMB settlement in cross-border trade. This underscores the important role of national strategic policies in advancing RMB internationalization, while also demonstrating the potential for synergy between enterprises and policy initiatives.

Heterogeneity analysis shows that the impact of export competitiveness on RMB settlement is more pronounced in certain contexts. For instance, smaller firms, with their flexible resource allocation and quick market responsiveness, tend to experience a stronger effect of competitiveness on RMB settlement. In highly concentrated industries, large leading firms play a crucial role in driving RMB settlement, amplifying the effect of competitiveness. Additionally, firms with higher operating liabilities, by reducing reliance on financial leverage, enhance their flexibility, further strengthening the role of competitiveness in promoting RMB settlement in cross-border trade.

Moreover, the increased use of RMB for cross-border settlements brings several positive spillover effects: (1) It significantly reduces firms’ dependence on imports, thereby improving supply chain resilience and risk management, contributing to the long-term stability of their competitiveness. (2) It enhances firm value, fostering a virtuous cycle between the capital markets and the real economy. This underscores the strategic significance of RMB settlement in enhancing firms’ global competitiveness and investment appeal.”

**6. variation between companies - scale and industry differences were not reflected in the analyses and interpretation process adequately.**

**Re**: Thank you for pointing out the need to account for variation between companies, particularly in terms of scale and industry differences. In response to your comment, we have incorporated an additional heterogeneity analysis focusing on firm size to reflect the varying impacts across companies of different scales. The relevant revisions are as follows, and the regression results are presented in the updated tables:

“4.5.1 Heterogeneity of firm size

To further investigate the heterogeneity of the impact of export competitiveness on RMB cross-border settlement, this paper conducts a subsample analysis based on firm size to reveal whether smaller or larger firms are more responsive to the advantages of RMB settlement when improving their export competitiveness.

To test this, firms are divided into two groups based on their total assets, using the median as the cutoff. Firms with total assets above the median are classified as large firms, while those below the median are considered small firms. Subsample regressions are then conducted for each group. The results are shown in columns (2)-(3) of Table 4. It can be seen that the effect of export competitiveness on RMB cross-border settlement is more significant in the small-firm group than in the large-firm group. It suggests that firm size plays a crucial role in moderating the relationship between export competitiveness and RMB cross-border settlement. Smaller firms, likely constrained by limited resources and bargaining power, are more sensitive to the benefits of RMB settlement when they enhance their export competitiveness. This highlights the importance of firm-level microeconomic factors in understanding currency choice in cross-border trade, particularly for smaller market participants.”

Additionally, we have conducted a more in-depth interpretation of the results for industry concentration differences as follows:

“4.5.2 Heterogeneity of industry concentration

The degree of industry concentration reflects the extent to which leading firms in the industry control the market, serving as a measure of the market structure of the industry. It indicates the level of competition and monopoly within the market. A high degree of industry concentration suggests the presence of a few large enterprises that dominate the industry’s development. These firms may have a pioneering role in driving export competitiveness and RMB cross-border settlement. Accordingly, this paper employs the industry Lerner index to measure industry concentration and divides the sample into two groups -high and low concentration- based on whether the index is above or below the median. Group regressions are then conducted.

The regression results are shown in columns (4)-(5) of Table 4. The coefficient for COMP is significantly negative in the high-concentration group, while it is not significant in the low-concentration group. The coefficient difference test between the two groups reveals a significant disparity, indicating that export competitiveness has a stronger effect on promoting RMB cross-border settlement in industries with higher concentration. This finding highlights the spillover effect of industry leaders in driving the expansion of RMB cross-border settlement across the entire industry.”

- **Reviewer #2:**

**Comment 1: Constructive Comments**

**1. At first glance, the aim, objectives, and methodology of the paper seem a bit vague. There are little adequate clarifications.**

Re: Thank you for your feedback regarding the clarity of the paper’s aim, objectives, and methodology. We have carefully reviewed and revised these sections to provide more explicit and detailed explanations.

First, we have refined the introduction to articulate the research aim more clearly, emphasizing the significance of examining how export competitiveness impacts RMB cross-border settlement at the firm level. Additionally, the objectives are now outlined more systematically to highlight their alignment with the research questions and theoretical framework.

Second, we have expanded the methodology section to include a more comprehensive description of the research design, data sources, and econometric techniques used. This update ensures that the analytical approach is transparent and demonstrates how the methodology supports the study’s objectives.

**2. In introduction section, the authors can reflect the relevant literature review in a more systematic way. In the current form, there is a great mess in the paper. In addition, the follow-up questions can be listed in the introduction to attract the attention of the readers.**

Re: Thank you for your valuable feedback on the introduction section. To address your concerns, we have made the following adjustments:

First, we have relocated the literature review from the introduction section to the theoretical analysis section. This change eliminates the clutter in the introduction and enhances the overall systematization of the paper. The theoretical analysis section now provides a more cohesive and comprehensive discussion of the relevant literature, aligning it directly with the study’s framework.

Second, we have added a research outlook at the end of the section to make the introduction more engaging and provide a clear structure. This outlines the follow-up questions and sets the stage for the subsequent analysis, thereby capturing the reader’s attention and providing a logical progression for the paper.

We believe these changes improve the clarity and organization of the paper and better meet the expectations of the readers. Thank you for highlighting this important aspect.

**3. I recommend to the authors to clarify their methodology in detail, making sure that their planned methods/research tools are fully detailed. They ought to give attention to justifying the chosen methodology in terms of demonstrating applicability, adjustment, and usefulness in the paper.**

Re: Thank you for your valuable feedback. In response to your suggestion, we have made several revisions to clarify and strengthen our methodology. The revisions in detail can be found in the revised manuscript.

First, we have provided a detailed description of the sample selection process, outlining the criteria and steps involved in choosing the companies for analysis. Additionally, we explain the rationale behind these decisions to ensure the sample’s representativeness and relevance to the research objectives.

Second, we have explicitly stated the methods used to calculate both the dependent and independent variables, ensuring transparency in how key metrics such as export competitiveness and RMB cross-border settlement are quantified. And we have also clarified the rationale for selecting control variables, with an explanation of why each variable was included and how it contributes to the robustness of the model.

Third, we have also enhanced our explanation of the model construction, emphasizing how the chosen methodology is applied to the data and how it aligns with the research objectives.

**4. Theory-practice nexus is a good tool for a “thesis vs. counter-thesis = synthesis” approach. In this context, the way how you support and proof your argument and falsify your counterarguments can be more effective to keep up being focused on your story. So that the central research questions and the “argument vs. counter-arguments interactions” are streamlined in a way that addresses the research inquiries in a systematic and effective manner.**

Re: Thank you for your insightful comment. In the *Theoretical analysis* section, we have strengthened the process of falsification by addressing a potential counterargument. Specifically, some may argue that macroeconomic factors are the primary drivers of firms’ RMB cross-border settlement in international trade. In response, we have provided both theoretical and practical refutations to this viewpoint, demonstrating that micro-level factors such as firm competitiveness also play a significant and positive role in promoting RMB cross-border settlement. This approach allows us to better structure our argument and counterarguments, ensuring a more focused and systematic presentation of the research inquiries.

**5. Referencing needs to be improved very carefully. The authors can enrich the literature review section through adding some up-to-date and relevant scientific materials.**

Re: Thank you for your constructive feedback. In response, we have carefully revisited the references in our paper and incorporated more up-to-date and relevant scientific materials, so to strengthen the foundation of our analysis and contribute to the overall quality of the paper.

**6. I strongly recommend the researchers to detect their scientific work via iThenticate / CrossCheck or Turnitin plagiarism software. Regarding the improvement of the quality of a scientific work in frame of scientific & ethical criteria, my advice to the authors would be to register to some free online interactive e-training courses which are listed as such: i) Making sense of science stories; ii) Publishers: Origins, roles and contributions; iii) The journal publishing cycle; iv) How do Editors look at your paper?; v) Preparing your manuscript; vi) Structuring your article; vii) Successful grant applications getting it right; viii) How to review a manuscript; ix) How to get published structuring your article; and x) Ethics responsibilities for Reviewers. The authors will learn about much more information (e.g., salami slicing, least publishable units, plagiarism, falsification, duplication, fabrication and so on) which are associated with preparing an excellent manuscript for a peer review journal.**

Re: Thank you for your valuable recommendation. In response to your suggestion, we have checked the manuscript for plagiarism using various tools, and the results indicate a low duplication rate. Additionally, we have actively engaged with resources on academic writing and research ethics to enhance the quality of the paper. We appreciate your guidance on the importance of ethical considerations in academic work, and we will continue to ensure that our research adheres to the highest standards. Thank you again for your constructive input, which has helped improve the manuscript.

**7. In fact, I appreciate there has been a lot of reading and ground covered, but this will not appeal to readers if the paper lacks a strong focus, compelling argument and discussion, and an indication of why the paper holds value to the readership of PLOS ONE.**

Re: Thank you for your insightful feedback. We understand the importance of maintaining a strong focus and a compelling argument throughout the paper. In response, we have made significant revisions to improve the focus and the overall argument of the paper. Specifically, in the *Introduction* section, we have strengthened the research objectives by clearly emphasizing the importance of micro-level competitiveness in influencing RMB cross-border settlement. We also provided concrete case studies, such as those of Midea and ZTO, to capture readers' attention and demonstrate the real-world relevance of the research.

Furthermore, in the *Theoretical analysis* section, we employed a more systematic approach by presenting both supporting and opposing arguments to deepen the theoretical discussion. This method not only enhances the logical flow but also strengthens the paper's contribution to the existing literature on international business and RMB internationalization. These revisions aim to ensure that the paper is not only compelling but also offers substantial theoretical and practical insights that are valuable to the readership of PLOS ONE.

**Comment 2: Some Technical Critiques and Recommendations for the Author**

**1. The author should strictly adhere to submission guidelines of the journal.**

Re: Thank you for your reminder. We have carefully reviewed and revised the paper to ensure full compliance with the journal’s submission guidelines.

**2. There are several grammatical errors and instances of badly worded/constructed sentences. Please check the manuscript and refine the language carefully. I suggest using proof reading papers before submission.**

Re: Thank you for your valuable feedback. We have thoroughly proofread the manuscript to address the grammatical errors and improve sentence structure. We have ensured that the language is clearer and more precise throughout the paper.

- **Reviewer #3:**

**Comment :**

**This paper attempts to address the issue concerning China’s efforts to internationalise its currency, Yuan. Good exports volume and export competitiveness promote a country’s own currency. In this case, China’s trade with the world constitutes a larger share of world exports, of which China is using its RMB to settle cross-border trade agreements. This also signifies the growing importance of China’s Renminbi (RMB) as an important currency of global trade and commerce.**

**The paper is analytic and informative, and stresses on the export competitiveness and the impact it has on internalisation of RMB. The authors have engaged in an analytical research and have used extensive datasets to test their hypothesis. It also stresses the role of currency in trade settlement across international borders. The authors have duly reviewed the literature and have drawn examples citing past studies on this topic.**

**The statistical analyses thus performed are robust, with multimodal framework designed to test the role of export competitiveness on promoting a nation’s currency. The theoretical analyses address both the microeconomic level and macro level picture depicting the choice of currency in cross-border trade settlement.**

**The authors have provided the datasets based upon which they have performed rigorous statistical analyses. They have also performed robustness test in line with the main objective of the paper to validate the hypotheses.**

**The authors firmly stuck to the core analysis to figure out what factors could strengthen cross-border used of RMB as means for settling cross- border trades and transactions. It has shed more knowledge on this frontier which could be taken up for further analysis to validate the theory of export competitiveness and how it promotes a country’s own currency, in this case, China.**

Re: Thank you for your positive feedback, and we appreciate your recognition of the analytical depth and the robustness of our statistical analysis.

We would like to thank all the reviewers and the editor for your detailed and constructive comments. We appreciate the time and effort you have put into reviewing our manuscript.

We believe that the revisions have strengthened the paper, particularly by improving the theoretical framework, expanding the empirical analysis, and refining the language to ensure clearer communication of our findings. We are confident that the revised manuscript is now more comprehensive and insightful, making a meaningful contribution to the literature on export competitiveness and the internationalization of the RMB.

Once again, thank you all for your careful and thorough review. We look forward to the possibility of our work being published in your esteemed journal.

Sincerely,

Lin Jiang

Southwestern University of Finance and Economics
